# Vessel architecture in human knee cartilage in children: an in vivo susceptibility-weighted imaging study at 7 T

**DOI:** 10.1007/s00330-017-5290-1

**Published:** 2018-02-26

**Authors:** Alexander Kolb, Simon Robinson, David Stelzeneder, Markus Schreiner, Catharina Chiari, Reinhard Windhager, Siegfried Trattnig, Klaus Bohndorf

**Affiliations:** 1 Department of Orthopaedic Surgery, Medical University Vienna, Vienna, Austria; 2 High Field MR Centre, Department of Biomedical Imaging and Image-guided Therapy, Medical University Vienna, Vienna, Austria

**Keywords:** Magnetic resonance imaging, Cartilage, Epiphyseal cartilage, Bone development, Osteochondritis dissecans

## Abstract

**Objectives:**

To evaluate the clinical feasibility of ultrahigh field 7-T SWI to visualize vessels and assess their density in the immature epiphyseal cartilage of human knee joints.

**Methods:**

7-T SWI of 12 knees (six healthy volunteers, six patients with osteochondral abnormalities; mean age 10.7 years; 3 female, 9 male) were analysed by two readers, classifying intracartilaginous vessel densities (IVD) in three grades (no vessels, low IVD and high IVD) in defined femoral, tibial and patellar zones. Differences between patients and volunteers, IVDs in different anatomic locations, differences between cartilage overlying osteochondral abnormalities and corresponding normal zones, and differences in age groups were analysed.

**Results:**

Interrater reliability showed moderate agreement between the two readers (*κ* = 0.58, *p* < 0.001). The comparison of IVDs between patients and volunteers revealed no significant difference (*p* = 0.706). The difference between zones in the cartilage overlying osteochondral abnormalities to corresponding normal zones showed no significant difference (*p* = 0.564). IVDs were related to anatomic location, with decreased IVDs in loading areas (*p* = 0.003). IVD was age dependent, with more vessels present in the younger participants (*p* = 0.001).

**Conclusions:**

The use of SWI in conjunction with ultrahigh field MRI makes the in vivo visualization of vessels in the growing cartilage of humans feasible, providing insights into the role of the vessel network in acquired disturbances.

**Key Points:**

*• SWI facilitates in vivo visualization of vessels in the growing human cartilage.*

*• Interrater reliability of the intracartilaginous vessel grading was moderate.*

*• Intracartilaginous vessel densities are dependent on anatomical location and age.*

**Electronic supplementary material:**

The online version of this article (10.1007/s00330-017-5290-1) contains supplementary material, which is available to authorized users.

## Introduction

The cartilaginous distal femoral epiphysis is penetrated by a complex canal network of blood vessels, showing successive stages in the development of the epiphysis [1–4]. As the ossification progresses the network of blood vessels within the cartilage diminishes, leaving the hyaline cartilage avascular at the end of growth [5, 6].

On the basis of the results of animal studies, focal impairment of vascularization, which leads to ischaemia of the epiphyseal cartilage during endochondral ossification, has been reported as one of the earliest events in the pathogenesis of osteochondritis dissecans (OCD) [7, 8]. The relatively high incidence of OCD in the human knee (6–10 per 100,000 patients) and potential need for surgery in OCD patients suggest the clinical importance of these intracartilaginous vessels [9–11]. The proposed use of susceptibility-weighted imaging (SWI) in conjunction with ultrahigh field MRI is a new approach to visualize vessels in vivo in the growing cartilage [12]. To the authors’ best knowledge, no previous studies have been conducted to quantify intracartilaginous vessels in immature patients and volunteers.

SWI is a magnetic resonance imaging (MRI) method that is used to image veins [13, 14]. Iron in haemoglobin gives rise to a small change in the magnetic field close to vessels which generates contrast between the vessels and the surrounding tissue in T2*-weighted imaging sequences. In contrast to most anatomical imaging in MRI, SWI uses the phase of the MR signal as well as the magnitude. To date, SWI has nearly exclusively been used in the brain. For human epiphyseal cartilage, there are only few reports which have discussed the potential of this method to gain knowledge about the distribution and development of vessels in vivo. In animal models it has been shown that vessels contained in cartilage canals can be identified ex vivo and in vivo using SWI in an experimental MRI scanner, which was confirmed by histological examination and μCT imaging [15]. Differences in distribution of vasculature between humans, pigs and goats could be demonstrated ex vivo in femoral epiphyseal cartilage [16, 17]. Using SWI Wang et al. could visualize abnormal vessels in areas of surgically induced osteonecrosis in juvenile goats [18]. The feasibility of SWI to image vessels and cartilage layers in juvenile human joints in vivo has recently been reported in a methodological study at 7 T [12]. In this study, we extend this prior work to assess the vascularity of juvenile epiphyseal cartilage in normal and diseased human knees using SWI in a human 7-T MRI scanner.

The aims of this study were

- To study the clinical feasibility of using ultrahigh field MRI using SWI to visualize vessels in the growing cartilage in the knee joint of healthy volunteers
- To assess the vascular pattern and density at different localizations in the knee joint
- To perform a preliminary cross-sectional clinical study in young patients with different clinically or radiologically suspected osteochondral disorders of the knee joint
- To conduct a preliminary analysis of the vascular pattern and density in different age groups

## Materials and methods

The study was approved by the ethics committee of our institution. Six volunteers (two female, four male) and six patients (one female, five male) participated in this study. Parents and legal guardians of children and adolescents, as well as the children and adolescents themselves, were informed about the possibility of participating in this study. Detailed information about the study aims and procedures was provided prior to recruitment, and participation was with written informed consent. Participants’ ages at the time of examination ranged from 6.8 to 15.2 years (mean age 10.3 years, volunteers) and 7.8 to 14.1 years (mean age 11.1 years, patients). The study was designed as a prospective, descriptive, case-based and cross-sectional.

The volunteer group (*n =* 6) consisted of patients of our department with trauma at sites other than the knee joint. All the knees investigated were asymptomatic with no history musculoskeletal disorders.

The patients (*n =* 6) were referred to us because of the following reasons: follow-up of retrograde drilling of OCD (patient 1); irregularities of the margins of the ossification centre of the femoral condyle seen in radiographs obtained after minor trauma or unspecific pain. At the time of referral these patients were asymptomatic (patients 2, 3, 5, 6); unspecific pain in the anterior knee joint and radiographs obtained showed a dorsal defect of the patella (patient 4).

### Imaging

The measurements of the knee joints were carried out with a whole body magnet 7-Tesla Siemens MRI system (Siemens Healthcare, Erlangen, Germany) and a 28-channel transmit-receiver coil (QED, Quality Electrodynamics LLC, Cleveland, OH).

SWI data was acquired with a 3D, T2*-weighted, gradient-echo scan with TE = 10.3 ms and repetition time (TR) = 23 ms, GRAPPA factor 2, receiver bandwidth of 140 Hz/pixel, with matrix sizes of 448 × 266–493 (the different matrix sizes in phase encoding direction were adjusted to subject-specific field of view (FOV)), 0.3 mm in-plane resolution and 1.0- to 1.4-mm-thick slices, acquisition time between 4 and 6 min. A low resolution short echo-time reference scan was acquired to allow phase images to be combined over channels with the COMPOSER method [19]. Combined phase images were Laplacian unwrapped, high-pass filtered, converted to a phase mask and multiplied four times by the magnitude to generate SWIs.

**Fig. 1:**
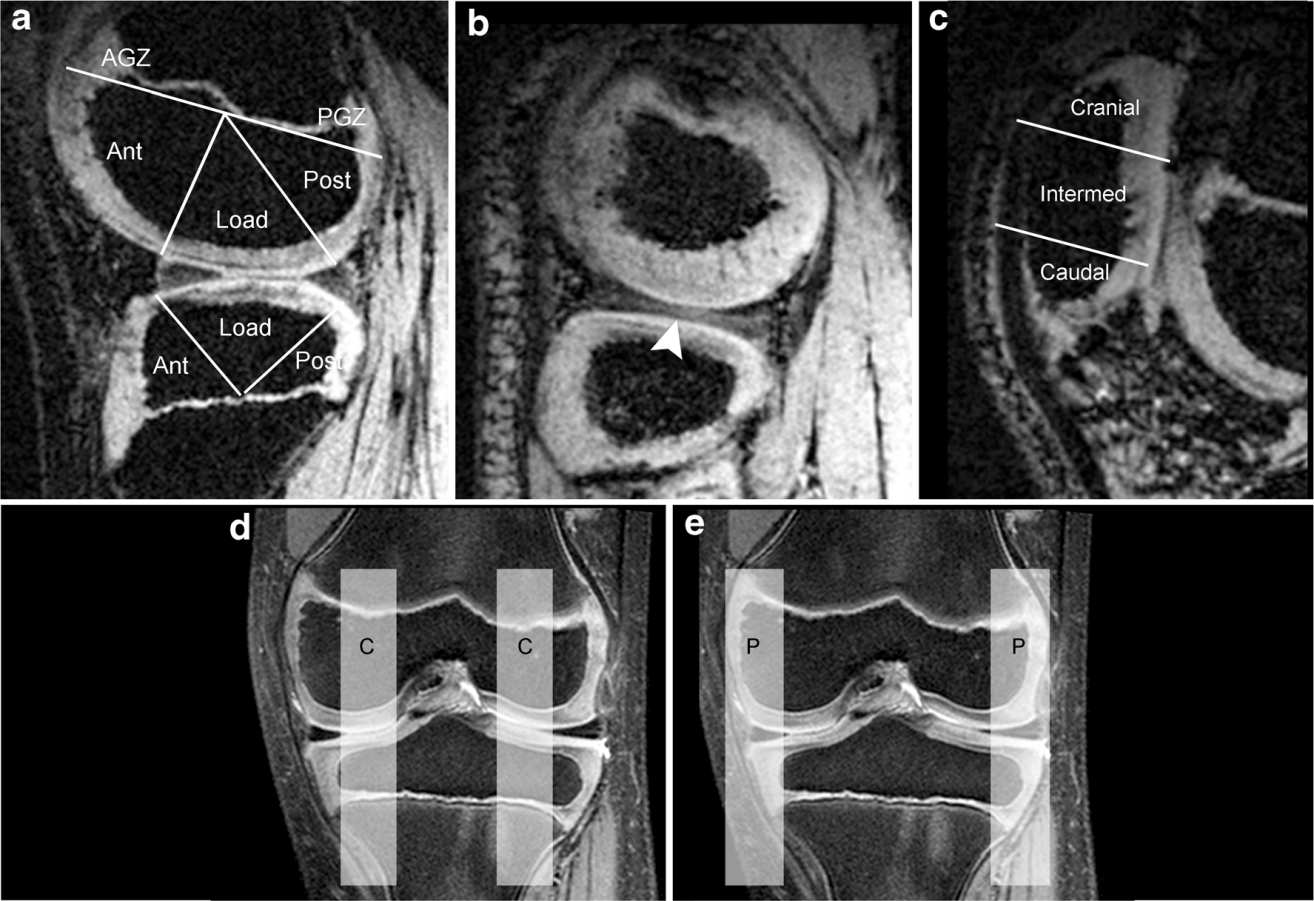
Definition of zones. **a** In the central sagittal plane of the medial and lateral femoral condyle five zones were defined, divided by a tangential line parallel to the growth plate and by two lines from the meniscal root to the centre of the growth plate: anterior growth zone (AGZ), anterior condyle (Ant), loading area (Load), posterior condyle (Post) and posterior growth zone (PGZ). In the tibial condyle three zones divided by lines from the meniscal root to the centre of the growth plate were defined: anterior condyle (Ant), loading area (Load), posterior condyle (Post). **b** The same zone definition was used in the peripheral sagittal slab, which was defined by a continuous meniscal shape (white arrow head), (example showing a lateral knee compartment). **c** In the patella three zones were defined by dividing the patella into thirds by two lines perpendicular to the patella axis: caudal (caudal), intermediate (intermed) and cranial (cranial) area. In the patella, only a sagittal slab of around 1 cm thickness in the central patella was analysed. **d** The coronal IMw morphological plane outlines the central sagittal slabs (C) and its location (hatched bars), as well as the peripheral sagittal slabs (P) in **e**

In six patients, morphological images were acquired with a fat-saturated intermediate-weighted (IMw) sagittal turbo spin-echo (TSE) sequence with a TE of 3500 ms and TE of 36 ms. The slice thickness was 2.4 mm. The interslice distance varied considerably because the automatic specific absorption rate (SAR) protection of the system did not allow a constant and clinically reasonable interslice distance of 10% of the slice thickness. As a consequence, a classical side by side comparison between the morphological images and the SWI images was not possible in all cases. The matrix sizes were 320 × 320–576 (the different matrix sizes in phase encoding direction were adjusted to subject-specific FOV). The GRAPPA factor was set at 2, and the acquisition time varied between 2 and 3 min. In one patient (patient 3) additional coronal images were obtained for illustration (Fig. 1).

### Image analysis

#### Susceptibility-weighted imaging

Image analysis was performed independently by two readers who were blinded to the participants’ identity, age and history. Zones were defined in the sagittal view for femoral and tibial condyles and for the patella on the basis of a modification of the classification by Peterfy et al. [20], as described in Fig. 1. In the lateral and medial femoral and tibial condyle, central and peripheral slabs were analysed separately. The slabs contained between 6 and 14 contiguous 1-mm slices dependent on the size of the knee.

The identification of vessels was based on SWI signal intensities as described by Nissi et al. [21], who demonstrated the concordance between SWI and vessel imaging using micro-CT and histological sections. Tools for vessel segmentation, which have been introduced in neuroimaging, are in the developmental stage [22] and do not reliably segment vessels in cartilage. For that reason to assess the size and number of vessels semi-quantitatively, “intracartilaginous vessel density” (IVD) in the epiphyseal cartilage was defined, which encompasses planar, punctuate and linear areas of hypointensity or no signal in the (high signal) cartilage.

**Fig. 2:**
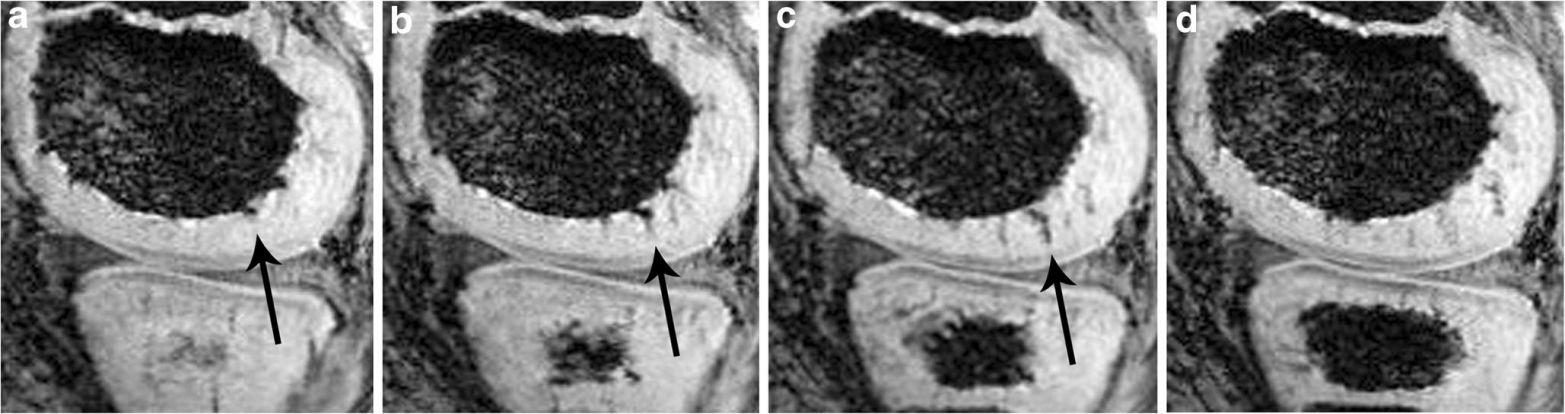
SWI of intracartilaginous vessels. Visibility and identification of intracartilaginous vessels by scrolling through the images (1 mm slices) of the slab in the sagittal plane: **a** the punctuate roundish area cannot be reliably identified as a vessel (arrow); however, in **b** and **c** the continuous slices identify the vessel with certainty (arrows). **d** The vessel is only faintly seen as a partial volume effect

These hypointense areas were partly round, partly geographic with irregular borders, showing a size of less than 2 mm. Both cancellous bone and slowly flowing blood in the veins lead to T2* dephasing and hypointensity. To avoid misinterpretation and overestimation of IVD at or very near the ossification front, the readers tried to establish whether hypointensities constituted contiguous structures which entered the epiphyseal cartilage when assessed over a number of the thin (1 mm) slices (Fig. 2).

**Fig. 3:**
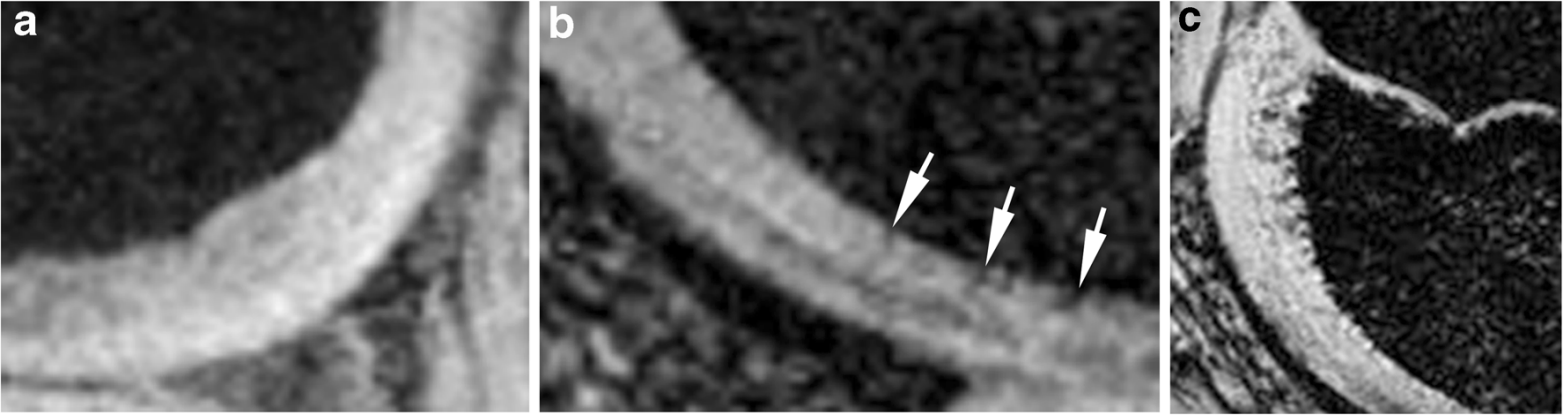
SWI of different parts of epiphyseal cartilage. White arrows show low signal intensities corresponding to intracartilaginous vessel formation in the femoral condyle. **a** Grade 0: no vessels can be identified. **b** Grade 1: some vessels can be seen near the ossification front as punctuate and linear areas of signal loss (arrows). **c** Grade 2: roundish, partly harmonic (“string of pearls”) representation of intracartilaginous vessels near the ossification front, clearly separated from the bone. The vessels become smaller closer to the loading zone

Areas about which there was uncertainty were not included in the rating. In each zone the central or peripheral slabs were summarized by the readers using the following definition (Fig. 3): grade 0 (no vessels), grade 1 (low density of vessels) and grade 2 (high density of vessels). These data (Table 1) were used for the following comparisons:

1. Patients versus volunteers: mean IVD in all regions in the femur, tibia and patella in patients compared to mean IVD of all regions in volunteers. Mean IVD of all regions of the femur, tibia and patella separately in patients and in volunteers.
2. Comparison of all IVDs (volunteers and patients) in the femur versus the tibia and the patella.
3. Comparison of the IVD of the lateral versus the medial femoral condyle in the volunteer group.
4. The IVD in areas with abnormal findings as morphologically defined was compared to the corresponding area of the opposite normal femoral condyle in the same knee.
5. The overall difference between the IVDs in the central slabs compared to the peripheral slabs of both femur and tibia was calculated.
6. The difference between the IVDs in the central loading area of the femur and the anterior and posterior femoral condyle was analysed using the data of all participants.
7. The overall (femoral, tibial, patellar) IVDs in three age groups were compared: 6–8 years old (group A), 8–11 years old (group B) and 11–15 years old (group C).

**Table 1:** Numerical ratings of intracartilaginous vessel density by two observers (given as rating of reader 1/rating of reader 2)

a	Lateral Femoral Condyle										Medial Femoral Condyle										b	Lateral Tibial Condyle						Medial Tibial Condyle						c	Patella		
	Central					Peripheral					Central					Peripheral						Central			Peripheral			Central			Peripheral						
	AGZ	Ant	Load	Post	PGZ	AGZ	Ant	Load	Post	PGZ	AGZ	Ant	Load	Post	PGZ	AGZ	Ant	Load	Post	PGZ		Ant	Load	Post	Ant	Load	Post	Ant	Load	Post	Ant	Load	Post		Caudal	Intermed	Cranial
Vol 1	2/2	2/2	1/1	2/2	2/2	2/2	2/2	2/2	2/2	2/2	2/2	2/2	1/2	2/2	2/2	-/-	-/-	-/-	-/-	-/-		2/2	1/1	2/2	2/2	2/1	2/2	2/2	1/1	2/2	2/-	2/-	2/-		2/2	1/2	2/2
Vol 2	2/2	2/2	2/2	2/2	2/2	2/2	2/2	2/2	2/2	2/2	2/2	2/2	1/1	2/2	2/2	2/2	2/2	2/2	2/2	2/2		2/2	1/1	2/2	2/2	2/2	2/2	2/2	1/1	2/2	2/2	2/2	2/2		2/2	2/2	2/2
Vol 3	2/2	1/1	0/1	1/2	2/2	2/2	2/1	1/1	1/2	2/2	2/2	2/2	0/1	2/2	2/2	2/2	2/2	2/2	2/2	1/2		2/2	0/0	1/2	1/2	1/1	1/2	1/2	0/1	1/2	2/2	1/1	2/2		1/1	0/1	1/2
Vol 4	2/2	2/1	1/1	2/2	2/2	2/2	2/2	2/1	2/2	2/2	2/2	2/2	1/1	2/2	2/2	2/2	2/2	2/2	2/2	2/2		2/2	0/1	1/1	2/2	0/1	0/1	2/2	0/1	1/2	2/2	1/1	2/2		1/2	0/1	2/1
Vol 5	0/0	0/0	0/0	0/0	0/0	0/0	0/0	0/0	0/0	0/0	0/0	0/0	0/0	0/0	0/0	0/0	0/0	0/0	0/0	0/0		0/0	0/0	0/0	0/0	0/0	0/0	0/0	0/0	0/0	0/0	0/0	0/0		0/0	0/0	0/0
Vol 6	1/2	1/2	1/1	1/1	2/2	2/2	2/2	2/1	2/2	2/2	1/2	2/2	0/1	1/2	2/2	1/2	2/2	2/1	2/2	1/2		2/2	0/1	1/1	2/2	2/1	2/2	1/2	0/1	1/2	1/2	1/2	1/2		0/2	1/1	1/2
Pat 1	1/0	0/0	1/0*	0/1	0/1	-/0	-/0	-/0	-/1	-/1	0/1	0/1	0/0	0/1	1/1	0/-	0/-	0/-	0/-	0/-		0/0	1/0	0/0	-/0	-/0	-/0	0/0	1/0	0/0	-/0	-/0	-/0		0/1	1/1	0/1
Pat 2	2/2	2/2	1/1	1/1	1/2	2/2	2/2	1/1	0/1	1/2	2/2	1/2*	0/1	1/1	2/1	2/2	2/2	2/1	2/1	2/1		1/2	1/1	1/1	1/2	1/1	1/1	1/2	0/1	1/1	2/2	1/1	2/2		0/1	0/1	1/1
Pat 3	2/2	2/2	1/1	1/2*	2/2	2/2	2/2	2/1	2/2	2/2	2/2	2/2	1/1	2/2	2/2	2/2	2/2	2/2	2/2	2/2		2/2	1/1	2/2	2/2	2/2	2/2	2/2	1/1	2/2	2/2	2/2	2/2		1/2	2/2	2/2
Pat 4	0/1	1/0	0/0	0/0	0/1	0/1	1/0	0/0	0/0	0/1	0/1	0/0	0/0	0/1	1/1	0/1	0/0	0/0	0/0	0/1		0/-	0/0	0/1	0/-	0/0	0/1	0/-	0/0	0/0	0/-	0/0	0/0		0/1	0/1*	2/1
Pat 5	2/2	2/2	1/1	1/2*	2/2	2/2	2/2	2/1	2/2	2/2	2/2	2/2	0/1	1/2*	2/2	2/2	2/2	2/2	2/2	2/2		2/2	1/1	2/2	2/2	2/2	2/2	2/2	0/1	2/2	2/2	2/2	2/2		2/2	1/2*	2/2
Pat 6	2/2	2/2	1/1	2/2	2/2	2/2	2/2*	2/2	2/2	2/2	2/2	2/2	1/1	2/2*	2/2	2/2	2/2	2/2	2/2	2/2		2/2	1/1	2/2	2/2	2/2	2/2	2/2	1/1	2/2	2/2	2/2	2/2		2/1	1/1	2/2

(a) Ratings in five anatomically defined areas in the central and peripheral parts of the lateral and medial femoral condyle (femoral anterior growth zone (AGZ), anterior (Ant), loading area (Load), posterior (Post), posterior growth zone (PGZ)). (b) Results of three areas at the medial and lateral tibial condyle (anterior (Ant), loading area (Load), posterior (Post). (c) Ratings of the central patella: caudal, intermediate (Intermed) and cranial area

Areas with pathologic findings in the cartilage or the cartilage below an osseous abnormality seen in morphological images are marked in bold (9 zones in a–c). Compare with “Supplementary analysis of morphological findings”

#### Morphological imaging

The morphological MR images of the patients were analysed by a radiologist with 30 years of experience in musculoskeletal radiology. The aim was to define the presence and the location of bony or cartilaginous abnormalities in the knee joint and to compare those areas with the same areas in the SWI. The analysis comprised the ossification centres and the epiphyseal or the hyaline cartilage of the femur, tibia and patella and used the same anatomic scheme as shown in Fig. 1.

### Statistical analysis

Data were processed using the SPSS 20 software (SPSS Inc., Chicago, IL, USA). Differences between the three age groups were analysed using one-way analysis of variance (ANOVA) and Tukey’s HSD (honestly significant difference) post hoc test for multiple group comparison. As defined by Landis and Koch, an interrater reliability analysis using weighted Cohen’s kappa coefficient was performed to determine the consistency of intracartilaginous vessel grading among raters [23]. Comparisons between patients and volunteers were made using *t* tests. The level of significance was defined as *p* < 0.05.

## Results

Image quality of the 7-T SWI scans was reproducible and excellent, regarding the absence of motion artefacts and clarity of anatomical structures.

### Analysis of SWI images

The numerical gradings of the IVD are listed in Table 1. Interrater reliability of the intracartilaginous vessel grading measured by Cohen’s kappa showed moderate agreement (*κ* = 0.58, *p* < 0.001).

**Fig. 4:**
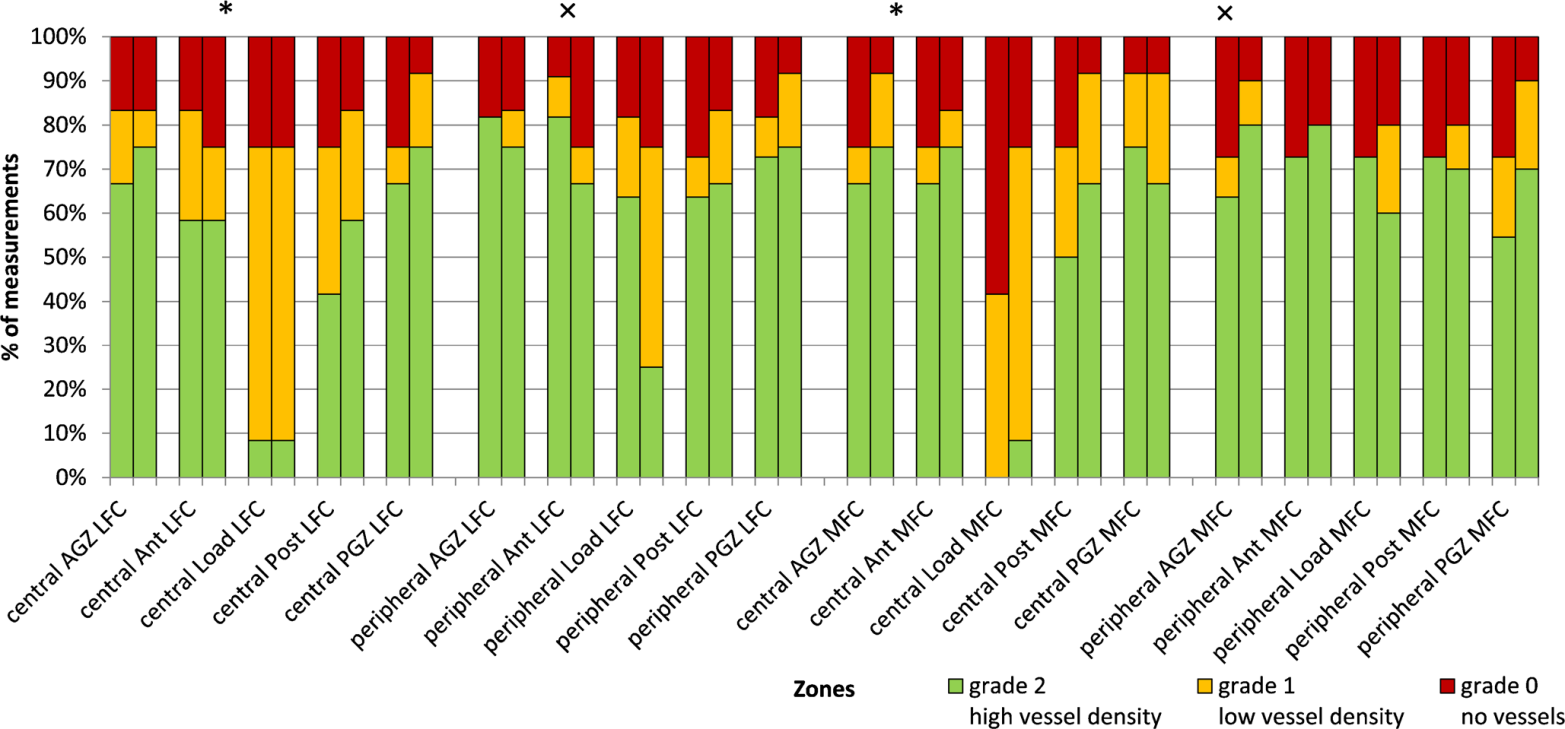
Femoral vessel density. Ratings of vessel density by the two readers in the lateral and medial femoral condyle (LFC and MFC; for definitions, see Fig. 1). Two adjacent bars are shown for each zone, with the ratings of reader 1 on the left and reader 2 on the right. A lower vessel density in the femoral central loading zones (central load LFC and central load MFC, marked by an asterisk above) can be observed compared to femoral zones adjacent anterior and posterior. This tendency cannot be clearly seen in the peripheral slabs (peripheral load LFC and peripheral load MFC, marked by a cross above). The femoral peripheral slabs show a higher vessel density compared to the central slabs. LFC lateral femoral condyle, MFC medial femoral condyle

**Fig. 5:**
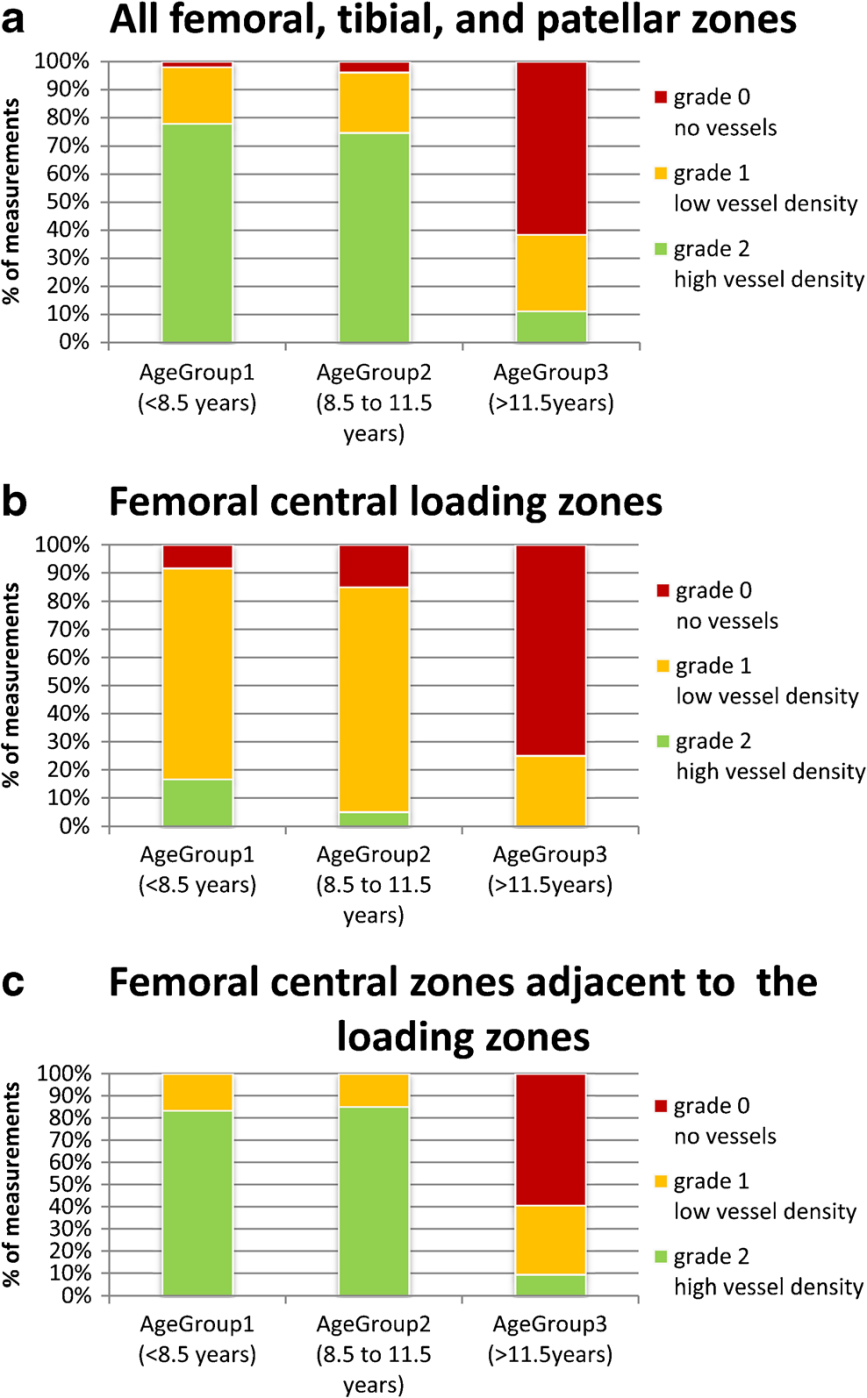
Grading of vessel density in different age groups within anatomically defined areas. Compared to the overall summary (**a**), differences between age groups differ in femoral loading zones (**b**) and femoral zones adjacent to the loading zones, showing that vessels tend to diminish earlier in loading zones and later in adjacent zones. **a** Summary of all measured femoral, tibial and patellar zones; **b** summary of the zones in all central loading areas of the medial and lateral femoral condyle; **c** summary of the zones in all femoral central areas anterior and posterior to the loading areas given in **b**. Number of volunteers and patients per age group (*n*): age group 1 (*n =* 3), group 2 (*n =* 5) and group 3 **(*n =*** **4)**

1. The overall IVD (including all tibial, femoral and patellar zones) was not statistically different between patients and volunteers (*p* = 0.706). Separating the analysis by region, the IVDs of tibia, femur and patella were also not statistically different between patients and volunteers (*p* = 0.827; *p* = 0.599; *p* = 0.878). The patient and volunteer groups did not statistically differ in age (*p* = 0.602).
2. In the pooled analysis over all participants, there were statistically non-significant differences between femoral and tibial zones (*p* = 0.086; mean femoral 1.29, and tibial 1.21) and femoral and patellar zones (*p* = 0.506; patellar mean 1.21).
3. There was no significant difference between the overall IVD in the lateral and the medial femoral condyle (*p* = 0.977, mean lateral 1.31, and 1.33 medial) in volunteers.
4. The preliminary intraindividual comparison of the IVD in zones with morphologically defined abnormal findings (see analysis of morphological images of patients in comparison to SWI) to the corresponding zone of the opposite normal femoral condyle in the same knee revealed no statistically significant difference (mean IVD 1.60 in both groups, *p* = 0.564).
5. Pooling controls and patients (*n =* 12), the overall difference between the measured IVDs in the central slabs compared to the peripheral slabs of both femur and tibia was significant (*p* = 0.016), with the density being higher in the periphery of the knee joints (mean IVD 1.41 versus 1.24).
6. The difference between the measured IVDs in the central femoral loading zones compared to the zones anterior and posterior to the loading zones was significant (*p* = 0.003), showing decreased values in the loading zones (mean IVD 0.73 versus 1.40) (Fig. 4).
7. The overall IVDs in the three age groups of the preliminary analysis are shown in Fig. 5. There was a significant difference in the mean IVD of all zones between the age groups, as determined by one-way ANOVA (*F* = 15.50, *p* = 0.001). However, a Turkey post hoc test revealed a significant difference between the age groups A and B compared to group C (*p* = 0.004, and *p* = 0.002). There was no statistically significant difference between the age group A and B (*p* = 0.986).

#### Analysis of morphological images of patients in comparison to SWI

Zones with pathologic findings in the cartilage or the cartilage below an osseous abnormality seen in morphological images are shown in Table 1 (nine zones, marked bold). The detailed analysis is given in the supplementary section “Analysis of morphological findings”.

## Discussion

Our feasibility study in 12 human patients and healthy controls shows that vessels with slowly flowing blood can be assessed semi-quantitatively in the epiphyseal growth cartilage of children using SWI in a human 7-T MRI scanner. Our study showed a moderate interrater reliability of the intracartilaginous vessel grading using a grading system based on three grades of IVD.

Further analysis showed that (1) IVDs depend on the anatomical location, (2) in the case studies, there was no apparent correlation between pathology and IVD, and (3) IVD decreases with age in different manners that are dependent on the anatomical location.

Although these findings were limited by the small sample size, our study suggests differences of IVDs related to the anatomical location in the following aspects: IVDs measured in the femoral condyles were slightly higher compared to tibial and patellar zones, but these differences were not statistically significant.

Our analysis of peripheral versus central femoral and tibial zones showed a significant difference with increased IVDs in the peripheral zones. Femoral loading zones were found to have a significantly lower IVD compared to the adjacent anterior and posterior zones.

This is, to our knowledge, the first time that this has been shown in vivo in humans. Tóth et al.’s group [16] showed the earlier regression of the central compared to the peripheral vascular supply in the femoral condyle in human cadavers. This is important, because in humans osteochondrosis dissecans develops preferably in the central areas of the medial femoral condyle [24, 25].

Our results also show that blood vessels in the epiphyseal growth cartilage are more frequent in younger age groups, but intracartilagenous blood vessels can still be detected in the age group of 11–15 years. This finding is in line with the results of Tóth et al. and Barnewolt et al. [26].

The analysis of the role of IVDs in areas overlying osseous abnormalities showed that there was no significant difference in the overall IVDs measured in the whole knee joint (epiphyseal cartilage of tibia, femur and patella) between patients and volunteers. This finding also held if the IVDs of tibia, femur and patella were evaluated separately. Moreover, no significant intraindividual differences were found in our six patients, comparing the IVD of the cartilage overlying the osseous abnormality with the IVD of the corresponding area of the opposite normal femoral condyle in the same knee.

Besides the small sample size of our cohort, there are several possible explanations for these negative findings. Firstly, differences in vasculature distribution, even if present, may not be detectable because SWI in our methodological setting did not allow sufficient spatial resolution and contrast-to-noise ratio to resolve an adequate number of small vessels. As shown, only a semi-quantitative estimation of the IVD was possible. Many of the differences between a clinical in vivo study and an experimental ex vivo or animal study using general anaesthesia lead inevitably to reduced image quality. Examination times in children have to be as short as possible to avoid or minimize motion artefacts. In our setting we used imaging parameters as described to achieve a measurement time between 4 and 6 min for the SWI sequence, which is short compared to the measurement times of 70 to 98 min used in experimental studies [16, 21].

Secondly, as a result of technical requirements of the investigational human 7-T scanner, only children with a body weight above 30 kg could take part in the study. Consequently patients and volunteers were relatively old (mean age 11.1 and 10.3 years). Our results indicate that at the age of 10 and above the amount of vessels in the residual growing cartilage detected on SWI is low, minimizing the chance to reveal differences of IVDs, even if present.

**Fig. 6:**
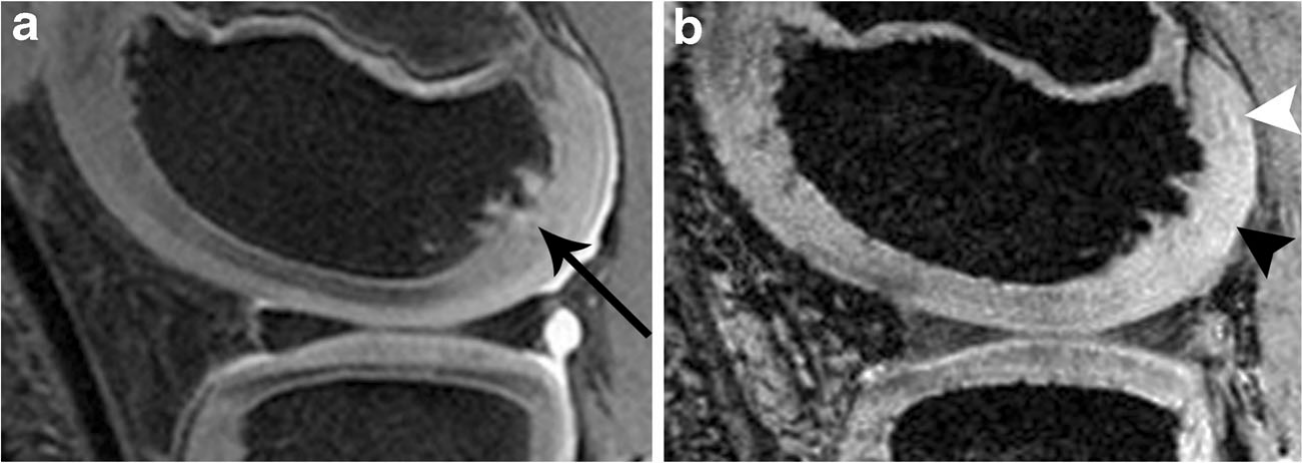
Ossification disturbance in the posterior part of the lateral femoral condyle. **a** IMw image with irregularities of the ossification centre (black arrow; see description in text). **b** SWI: the epiphyseal cartilage directly overlying the lesion shows a reduced density of vessels (black arrowhead, grade 0) while more proximally some vessels can be delineated faintly (white arrowhead). Also compare with the anterior part of the femoral condyle

Thirdly, the patient selection probably was not well suited to revealing differences in IVDs of the epiphyseal cartilage between controls and patients. Severely injured patients or patients with severe disease (e.g. septic arthritis) were not part of our study protocol. The majority of our patients (5/6) only had minor trauma or pain and were evaluated because of roentgenological irregularities of the border of the ossification centre. It was assumed that the osseous changes seen in roentgenograms may be accompanied by relevant cartilaginous changes over the osseous abnormalities. However, as revealed by morphological MRI these irregularities were due to not yet ossified, probably cartilaginous, parts in the bone, while the cartilage overlying the osseous lesions did not show any abnormalities, neither morphologically nor obvious changes of the IVD as revealed by SWI. Only in one anecdotal case (Fig. 6) we had the impression that the IVD was diminished below the osseous abnormality. Patient 1 with proven OCD was 15 years old and did not show relevant vasculature in the cartilage, neither in normal parts nor in the cartilage overlying the OCD.

Nonetheless, the included patients provide an insight into possible clinical applications and related limitations. As a consequence, recruitment criteria for further studies should focus on younger patients including distinct osteochondral pathologies, as the weight limit of human 7-T scanners might be decreased in the future. However, as OCD is rare in young patients, irregularities of the border of the ossification centre are an interesting phenomenon in young patients. Anyhow, the role of reduced blood supply in these irregularities, which seems to be crucial in the pathogenesis of OCD [27], as well as the relation of such irregularities to OCD, remains unclear.

## Conclusions

The use of susceptibility-weighted imaging (SWI) in conjunction with ultrahigh field MRI is feasible to visualizing vessels in vivo in the growing cartilage of humans. SWI shows potential for the depiction of differences in vessel density of the epiphyseal growth cartilage, related to age and anatomic location. This study did not demonstrate differences in vessel densities between healthy volunteers and patients with osseous ossification disturbances.

## Electronic supplementary material

ESM 1 (DOCX 3265 kb)

ESM 2 (DOCX 15 kb)

## Abbreviations

IVD: Intracartilaginous Vessel Density
OCD: Osteochondritis Dissecans
SWI: Susceptibility-Weighted Imaging
