# Supplementary material for: Vessel architecture in human knee cartilage in children: an in vivo susceptibility-weighted imaging study at 7 T
Source: Eur Radiol. 2018 Feb 26;28(8):3384–92. doi: 10.1007/s00330-017-5290-1 (PMC6028839; doi:10.1007/s00330-017-5290-1)
Supplement: Supplementary file 1 — (DOCX 3265 kb) [file 330_2017_5290_MOESM1_ESM.docx]

**
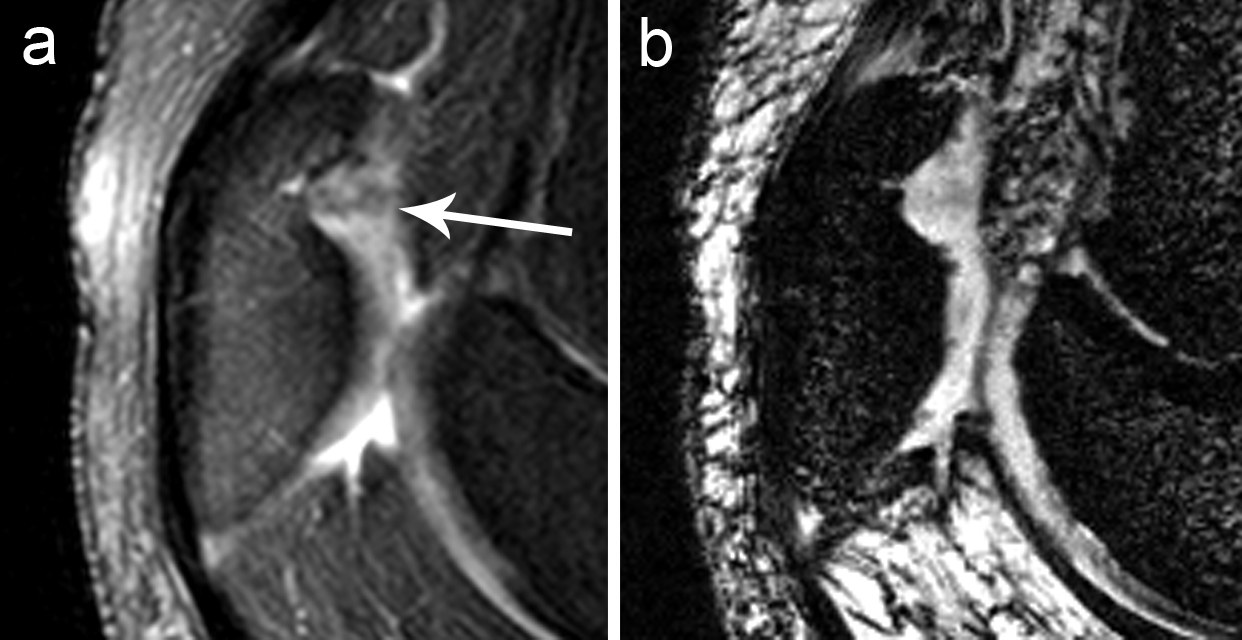
**

**Supplementary Fig. 1: Irregularity of the ossification center of the patella.** (**a**) IMw TSE: Irregularity of the contour of the ossification center of the patella showing epiphyseal cartilage without morphological abnormality (white arrow). (**b**) SWI: No vessels are detected within the irregularity.

**
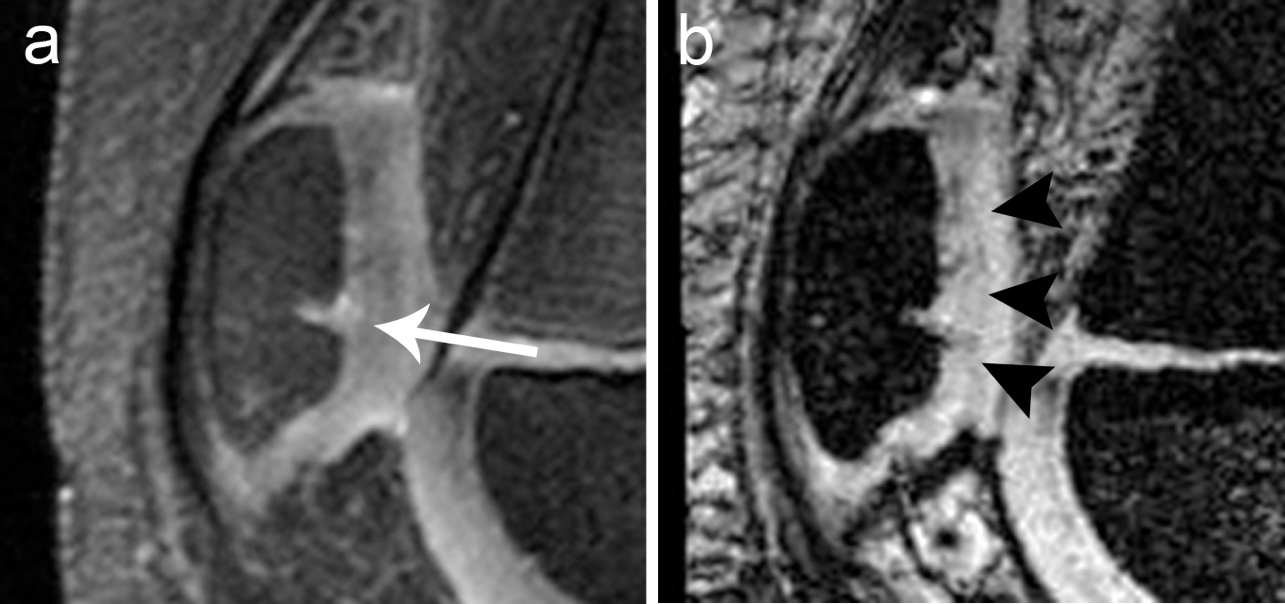
**

**Supplementary Fig. 2: Irregularity of the ossification center of the patella.** (**a**) IMw TSE: Irregularity of the contour of the ossification center of the patella (white arrow). The high signal area probably represents non organized cartilaginous tissue. (**b**) SWI: There is no obvious reduction of the intracartilaginous vessel density in the vicinity of the lesion (black arrowheads).
