# Supplementary material for: Vessel architecture in human knee cartilage in children: an in vivo susceptibility-weighted imaging study at 7 T
Source: Eur Radiol. 2018 Feb 26;28(8):3384–92. doi: 10.1007/s00330-017-5290-1 (PMC6028839; doi:10.1007/s00330-017-5290-1)
Supplement: Supplementary file 2 — (DOCX 15 kb) [file 330_2017_5290_MOESM2_ESM.docx]

***Analysis of Morphological images of patients (n=6) in comparison to SWI***

In all patients there were no abnormal findings regarding intraarticular or extraarticular pathology (ligaments, menisci, and peripheral soft tissues) besides the osseous and cartilaginous changes described below.

**Patient 1**

This 14 year old patient came for follow-up after retrograde drilling of an osteochondritis dissecans. The epiphyseal plate of the femur was closed and there was no epiphyseal cartilage. The lesion was in the central loading zone of the lateral femoral condyle. There was a high signal area between the cartilage and cancellous bone and a broader ring of edematous reaction. The cartilage was undulated but did not reveal any discontinuity. The signal of the cartilage beneath the lesion was partly reduced. SWI did not reveal abnormal vessels in the hyaline cartilage beneath the OCD.

**Patient 2**

This 12 year old patient had a pathological irregularity of the border of the ossification center in the central medial femoral condyle anteriorly. The irregularities in the bone consisted of a band-like, partly irregular area of high signal. The epiphyseal cartilage directly beneath the lesion was morphologically normal and showed a normal density of vessels.

**Patient 3**

This 7 year old patient had a pathological irregularity of the border of the ossification center in the lateral central femoral condyle posteriorly. The irregularities in the bone consisted of partly roundish, partly irregular small areas of high signal. Directly beneath the lesion the epiphyseal cartilage itself was morphologically normal but showed a reduced density of vessels (Grade 0) while more proximal the density was higher (Fig. 6).

**Patient 4**

The patient was 11 years old. In the intermediate part of the patella a pathological irregularity of the border of the ossification center was delineated. The irregularities in the bone consisted of partly roundish, partly irregular small areas of high signal. Directly beneath the lesion the narrow epiphyseal cartilage showed no morphological abnormality and no vessels (see Supplementary Fig. 1).

**Patient 5**

This 8 year old patient had a pathological irregularity of the border of the ossification center in the medial and lateral femoral condyle posteriorly. In both condyles the central slab was involved. Also the patella showed some irregularity (Supplementary Fig. 2). The SWI images around the lesion in these 3 regions did not reveal obvious abnormalities of the vessel density. In all cases some vessels could be identified in the vicinity of the osseous abnormalities.

**Patient 6**

This 11 year old patient had minor contour irregularities of the ossification center in the medial femoral condyle posteriorly and in the lateral condyle anteriorly. On the lateral side the peripheral slab and on the medial side the central slab was involved. The SWI around the irregularities did not reveal obvious abnormalities of the vessel density.
